# Predictive biomarkers and preventive strategies for PTLD: a turning point in viro-immunologic precision medicine?

**DOI:** 10.3389/ti.2026.16305

**Published:** 2026-05-04

**Authors:** Sophie Caillard, Morgane Solis, Ilies Benotmane, Floriane Gallais, Samira Fafi Kremer

**Affiliations:** 1 Department of Nephrology Dialysis and Transplantation, Strasbourg University Hospital, Strasbourg, France; 2 Molecular ImmunoRhumatology, INSERM UMR_S1109, Strasbourg University, Strasbourg, France; 3 Department of Virology, Strasbourg University Hospital, Strasbourg, France

**Keywords:** biomarker, EBV, HSCT, PTLD, SOT (solid organ transplant)

## Abstract

Post-transplant lymphoproliferative disorders (PTLD) represent a heterogeneous group of complications with rising complexity, particularly as EBV-negative forms are increasingly recognized in adult recipients. While early EBV-driven PTLD was historically monitored by viral load, this approach fails to detect EBV-negative disease and lacks specificity even in EBV-positive cases. Recent advances in tumor immunology, virology, and liquid biopsy technologies have led to the emergence of novel biomarkers that offer improved diagnostic precision. These include plasma soluble ZEBRA antigen, reflecting lytic EBV activation; EBV DNA methylation status, which may distinguish latent from benign viral replication; and LMP1 sequence variants that influence immune evasion through the NKG2A/HLA-E axis. For EBV-negative PTLD, circulating tumor DNA profiling has shown promise for early, non-invasive detection. These innovations are complemented by preventive strategies such as anti-CD20 therapy in high-risk EBV-seronegative transplant recipients and ongoing trials of EBV-targeted vaccines. However, such approaches remain limited to EBV-naïve patients. Moving forward, integrating viral, immune, and tumor-derived markers—alongside host genetic factors—may enable more personalized surveillance and preemptive interventions. This review outlines the evolving paradigm of PTLD monitoring and highlights key areas where viro-immunologic precision medicine may reshape clinical practice.

## Introduction

Post-transplant lymphoproliferative disorders (PTLD) occupy a unique space in transplantation medicine: relatively rare, with an incidence reaching two percent after 10 years in solid organ recipients [1], but disproportionately feared because of their unpredictability, severity, and the inadequate precision of available monitoring tools. For decades, PTLD was predominantly an Epstein–Barr virus (EBV)-driven complication occurring early after transplantation [2, 3], detectable in some cases by rising EBV viral loads [4], especially in paediatric population [5]. This paradigm has been replaced by a much more complex picture, shaped by the rise of EBV-negative PTLD occurring later after transplantation and by deeper insights into the immune dysfunctions underlying both EBV- and non–EBV-driven lymphoproliferations. Moreover, improved understanding of the pathophysiology of this malignant process may support the development of a new generation of biomarkers that are more specific than those currently used, which are often unreliable.

## Epidemiological shift towards EBV-negative PTLD

The first notable shift to highlight is epidemiological. In a recent Canadian registry study, the authors reported that adult solid organ transplant recipients now face a sustained risk of late-onset PTLD, whereas the risk of early PTLD appears to have declined in recent years [6]. Multiple cohorts, including the French K-VIROGREF registry, now report that EBV-negative PTLD represents nearly half of all cases of PTLD in adults. This is a profound transformation, because EBV-negative disease is biologically distinct from EBV-positive forms.

## Divergent pathways in EBV-negative versus EBV-positive PTLD

This observation implies that viral monitoring may fail to detect a substantial proportion of lymphoproliferative disorders. In other words, half of PTLD may be virologically silent and immunologically invisible unless new classes of biomarkers are developed. Instead of uncontrolled expansion of EBV-infected B cells due to defective immune surveillance, EBV-negative PTLD exhibits genomic instability and molecular hallmarks close to *de novo* diffuse large B-cell lymphoma, including recurrent TP53 pathway alterations, overexpression of DNA interaction-related genes and a heavy burden of chromosomal copy-number changes [7, 8]. Pathology studies in tumors retrieved from solid organ transplant recipients have further shown that the immune microenvironment of EBV-negative PTLD is characterized by CD4-positive T cells expression of exhaustion markers such as TIM-3 [9]. Moreover, NK cells demonstrate also dysfunctional phenotypes, and tumor infiltration by effector lymphocytes is sparse [9].

**FIGURE 1:**
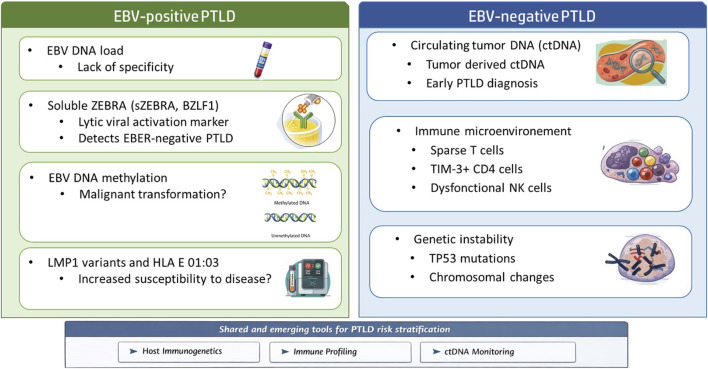
Predictive biomarkers for EBV-positive and EBV-negative PTLD.

Simultaneously, advances in the understanding of EBV-positive PTLD have shed new light on its complex pathogenesis. Far from a simple failure of CD8-mediated control, EBV-positive PTLD now appears to emerge from a confluence of factors. Dysfunctional NK cells, despite phenotypic activation, lack cytotoxic competency; CD8-positive T cells demonstrate impaired effector programs; and the microenvironment is enriched in PD-L1 expression, offering an immune-escape niche [9]. These insights have inspired the search for biomarkers that look beyond viral quantity to viral behavior, viral genetics, and the host’s capacity to respond Figure 1.

## Innovative biomarkers and PTLD risk assessment

### Soluble ZEBRA: a marker of lytic EBV activation

One promising development is soluble ZEBRA (Z Epstein-Barr virus replication activator, a protein encoded by the EBV *BZLF1* gene), a marker of lytic viral activation that appears more specific than the classical EBV viral load for EBV-driven PTLD [10, 11]. The presence of ZEBRA in plasma likely reflects a biologically meaningful transition from viral latency to lytic activation—an early event associated with oncogenic reprogramming. Moreover, studies have shown that proteins expressed during the lytic cycle contribute not only to cellular transformation but also to subsequent tumor development in animal models and human EBV induced tumors [12]. Indeed, modulation of host gene expression by ZEBRA can also deregulate the immune surveillance, allow immune escape, and favor tumor progression [13]. In a recent multicentric cohort of solid organ and hematopoietic stem cells transplant recipients, soluble ZEBRA achieved specificity over ninety percent and sensitivity above eighty percent for EBV-positive PTLD, outperforming EBV viral load and, surprisingly, identifying four cases of EBER (Epstein-Barr virus-encoded small RNA) negative tumor with negative blood EBV PCR assays [10]. The performance of this test was lower in an external validation cohort of solid organ and hematopoietic stem cells transplant recipients [11], but the accuracy of this biomarker still needs to be refined and assessed in additional cohorts of solid organ transplant recipients.

### EBV DNA methylation as diagnostic tools

A parallel innovation comes from epigenetic analyses, particularly DNA methylation assays. DNA methylation plays a central role in the regulation of gene expression, and both host genomic DNA and EBV DNA methylation are emerging as potential biomarkers. Two patients with the same EBV viral load may have different viral infections. A highly methylated EBV signal would suggest latent EBV within tumor cells, whereas unmethylated DNA could indicate a benign lytic infection. Recently, plasma EBV DNA methylation profiles were shown to discriminate three distinct EBV-associated conditions—infectious mononucleosis, EBV-associated lymphoma, and nasopharyngeal carcinoma [14]. In a small study conducted in patients who had undergone hematopoietic stem cell transplantation, one patient with predominantly unmethylated EBV DNA failed to respond to rituximab, whereas those with highly methylated EBV patterns were consistent with PTLD and responded to anti-CD20 therapy [15]. Beyond EBV DNA, host genomic DNA methylation status is also of major interest in oncology. Malignant tumors are characterized by aberrant DNA methylation. Panels of methylated host genes in circulating cell-free DNA (cfDNA) have also shown potential as diagnostic and prognostic biomarkers in EBV-associated lymphomas in non-transplant patients [16].

### Genomic characterization and immune evasion pathways

Another breakthrough lies in the genomic characterization of EBV itself. The risk of EBV-associated PTLD has recently been linked to the NKG2A/LMP1/HLA-E axis, involving specific viral and host variations [17]. More specifically, recent sequencing analyses have identified two variants of the EBV LMP1 gene—GGDPHLPTL and GGDPPLPTL—that are markedly enriched in solid organ and hematopoietic stem cells transplant recipients who develop EBV-positive PTLD. Moreover, patients with PTLD more frequently carry the HLA-E*0103/0103 allele, indicating an increased susceptibility to disease when EBV peptides are presented in this context. Lastly, the GGDPHLPTL and GGDPPLPTL LMP1 peptides strongly inhibit NKG2A^+^ NK cells and CD8^+^ T cells, thereby promoting immune evasion by these EBV strains. In the same manner, in a cohort of pediatric solid organ transplant recipients, the presence of two specific mutations in the EBV LMP1 gene (G212S and S366T) was associated with a nearly 12-fold increased risk of developing PTLD. Nevertheless, it is important to note that these mutations were also found at a high frequency in patients without PTLD; however, the absence of these mutations was associated with a high negative predictive value for PTLD occurrence [18]. Although these findings have been described mainly in young patients, they raise the possibility that EBV sequencing could become part of transplant risk assessment, with EBV lineage analysis helping to predict which patients are most vulnerable to malignant transformation following primary EBV infection.

### Liquid biopsy strategies

While EBV-focused biomarkers continue to advance, the detection of EBV-negative PTLD may benefit most from technologies adapted from oncology, particularly by detection of circulating tumor DNA. CfDNA sequencing studies have demonstrated that tumor-derived DNA fragments—harboring lymphoma-associated mutations and characteristic copy-number changes in specific genes—can be detected in plasma months before clinical diagnosis. In one landmark study, cfDNA levels were increased not only in solid organ transplant recipients with EBV-positive PTLD but also in those with EBV-negative PTLD, with the observed range of cfDNA levels being consistent with that reported in other malignancies [19]. Moreover, cfDNA levels correlated with tumor burden. Beyond genomic cfDNA profiling, cfDNA methylation provides an additional biomarker. To date, cfDNA methylation has not yet been evaluated in PTLD. However, similar to EBV-positive lymphomas, aberrant methylation patterns in cfDNA can be used as diagnostic biomarkers and may enable earlier cancer detection -including lymphoma-than mutation-based cfDNA assays [20]. In addition to their diagnostic value, these methylation patterns have also shown prognostic significance, being associated with clinical outcomes [21]. The implications are substantial: EBV-negative PTLD, once thought to be “invisible” until symptoms arise, may in fact shed detectable genetic material long before overt disease. For clinicians, this could offer a reliable tool for pre-symptomatic detection, enabling diagnosis at an early stage when treatment is more effective and potentially improving patient outcomes. However, despite these promising results, data correlating EBV DNA methylation patterns with EBV load dynamics or response to rituximab remain limited, and validation in larger cohorts is still required.

### Torque teno virus load as a surrogate marker of global overimmunosuppression

Torque teno virus (TTV) load has emerged as a marker reflecting the net state of immunosuppression in solid organ and hematopoietic stem cell transplant recipients. Although high TTV titers have been associated with infectious complications, current evidence does not establish a clear relationship between TTV replication and PTLD risk. Only a small exploratory study have suggested a possible association between high TTV viral load and the development of PTLD in hematopoiectic stem cell transplant patients [22], and no validated thresholds exist to date to guide PTLD prediction.

## Emerging preventive strategies for PTLD

In parallel, preventive strategies for PTLD are advancing. The REPLY randomized trial evaluating preventive rituximab in EBV D+/R–kidney transplant recipients has completed enrolment and may soon clarify whether early B-cell depletion can prevent primary EBV infection and subsequent PTLD in treated patients (NCT04989491). Another promising development is the emergence of EBV vaccines, including mRNA- and nanoparticle-based platforms, which are currently being evaluated in healthy young adults with the aim of reducing the incidence of infectious mononucleosis (NCT05164094, NCT05831111, NCT06908096). If effective, these vaccines could substantially lower the burden of EBV-driven PTLD in EBV-seronegative transplant recipients and potentially reshape clinical practice. However, both strategies—anti-CD20 therapy and EBV mRNA vaccination—would primarily benefit patients who are EBV seronegative, and therefore would not apply to the majority of adult transplant recipients, who are mostly EBV seropositive. Conversely, these approaches may prove particularly valuable in paediatric transplantation, where a large proportion of candidates are EBV-naïve and at high risk for primary infection and early PTLD development.

## Conclusion

In conclusion, significant hope has emerged from preventive strategies—either vaccination or therapies aimed at depleting the EBV reservoir—but these approaches apply primarily to EBV seronegative organ recipients. In parallel, there is a clear need to refine biomarkers that can identify patients at highest risk of developing PTLD, whether during primary EBV infection or later, at the time of viral reactivation. In addition, specific biomarkers for EBV negative lymphomas—which are becoming increasingly frequent due to the aging transplant population—must also be developed. A combination of genetic data, associated with virological, immunological, and tumor-derived markers could enable a more effective and personalized approach to PTLD screening, whether EBV-driven or not. Among these emerging candidates, HLA-E genotyping, plasma ZEBRA protein measurement, viral DNA methylation, sequencing of the viral LMP1 protein, and circulating tumor DNA quantification represent particularly promising opportunities.
